# Recent Advances and Future Challenges in the Field of Digestive Diseases

**DOI:** 10.3390/medicina59020208

**Published:** 2023-01-20

**Authors:** Ludovico Abenavoli, Marcello Candelli

**Affiliations:** 1 Department of Health Sciences, University “Magna Graecia”, Viale Europa—Germaneto, 88100 Catanzaro, Italy; 2 Department of Emergency Medicine, Fondazione Policlinico Universitario “A. Gemelli” IRCCS, L. go A. Gemelli 8, 00168 Roma, Italy

Digestive diseases are a rapidly evolving area of clinical and research. New technologies, novel therapies, and better knowledge of pathogenetic mechanisms are the main drivers of this growth [1]. However, some aspects of gastroenterology and hepatology remain insufficiently investigated and can be considered unmet medical needs that require solutions in the near future [2]. Digestive diseases comprise a multidisciplinary and interdisciplinary area of research, with many clinical aspects focused on specific organs, all of which are investigated multiple unique functional and morphological diagnostic investigations and several auxiliary disciplines, such as biochemistry, pathological anatomy, physiology, cell biology, neuroendocrinology, neuro-gastroenterology, immunology, molecular biology and genetics [3]. Moreover, there has recently been rapid development in the field of endoscopy. Complex invasive procedures are gaining visibility, and the number of potential applications is expanding. The challenge in the near future will be to balance the appropriateness of gastrointestinal endoscopy with clinical indications and the evolution of techniques [4].

The field of digestive diseases has developed considerably in recent decades due to the fruitful research of eminent scholars and researchers, which originally allowed for improvements in our knowledge of physiological and anatomical changes in various aspects of the gastrointestinal tract, some of which remain the subjects of study and development today [5]. For example, it is worth highlighting the progress made in the study of gastric acid secretion and the mechanisms that control it at the cellular level. As a result of this research, new, effective drugs with anti-secretive activity were studied and subsequently commercialized, with a consequent drastic reduction in the use of surgery to treat acid-related diseases of the upper digestive tract [6]. In addition, improved knowledge of digestive tract motility has motivated several studies that have elucidated the central role of the gastrointestinal autonomic nervous system and visceral sensitivity in various functional disorders, from the esophagus to the colon [7].

The role of infectious agents as causes of major gastrointestinal diseases has also been documented, particularly the role of Helicobacter pylori infection as the most important pathogenic factor associated with the occurrence and development of peptic ulcers. In 2005, the Nobel Prize in Physiology or Medicine was awarded to Barry Marshall and Robin Warren, which is one of the rare examples of this prestigious prize being given to clinical scholars in the field of medicine [8]. These extraordinary discoveries have led to the development of innovative and effective new therapies, such as the initial development of H2 antagonists and the later development of proton pump inhibitors and their combination with antibiotics, which have made it possible to successfully—and often definitively—treat many patients with gastrointestinal diseases [9].

The disease spectrum of non-alcoholic fatty liver disease encompasses a wide range of patients and represents a public health burden of epidemic proportions [10]. Findings from nutrigenetics and nutrigenomics suggest the capability of nutrition to influence the clinical outcome of these patients [11]. Emerging pharmacologic strategies for the treatment of hepatic steatosis will be multi-pronged and target metabolic pathways including insulin sensitivity, oxidative stress, and fatty acid synthesis [12]. This is a burgeoning and hopeful field of study. The perspectives are bright for clinicians and researchers engaged in the study and treatment of non-alcoholic fatty liver disease.

In recent decades, the diagnosis of digestive diseases has also benefited from endoscopy and its technological advancements, which have allowed gastroenterologists to make a significant leap in the quality of diagnosis. These new technologies have strengthened the role of the clinical gastroenterologist and helped to characterize gastroenterology in every aspect as a medical specialty in its own right [13].

In addition, the recent integration of artificial intelligence into gastroenterology and hepatology will transform the field of digestive diseases in the coming years [14]. The aim is to reduce the time spent on documentation and maximize the time spent with the patient, which remains the ultimate goal of any medical practice. Present and future gastroenterologists will face new challenges in the way that health care is delivered, driven by economic and demographic changes, social trends, technological innovations, and scientific advancements.

At the end of this analysis, we can say that digestive diseases comprise a living discipline capable of impressive future developments. We strongly believe that further innovations will help specialists to apply more personalized treatments tailored to specific diagnostic procedures and therapies. We believe that, in line with recent technological advancements, including artificial intelligence and new endoscopic technologies, gastroenterologists will provide patients with new tools to improve the appropriateness and quality of treatments. In this context, we strongly believe that as gastroenterologists, we will enhance and strengthen partnerships with many other specialties, such as nutrition, diabetology, surgery, and internal medicine, to provide integrated, multidisciplinary care. Finally, myriad areas of basic and applied research can bring great satisfaction to young physicians approaching this specialty, which remains one of the most coveted in the Western world.
